# Toward a Clinical Definition of Early Osteoarthritis: Onset of Patient-Reported Knee Pain Begins on Stairs. Data From the Osteoarthritis Initiative

**DOI:** 10.1002/acr.22418

**Published:** 2014-12-27

**Authors:** Elizabeth M A Hensor, Bright Dube, Sarah R Kingsbury, Alan Tennant, Philip G Conaghan

**Affiliations:** 1 Leeds Institute of Rheumatic and Musculoskeletal Medicine, University of Leeds, and NIHR Leeds Musculoskeletal Biomedical Research UnitLeeds, UK; 2 Leeds Institute of Rheumatic and Musculoskeletal Medicine, University of LeedsLeeds, UK

## Abstract

**Objective:**

Early detection of osteoarthritis (OA) would increase the chances of effective intervention. We aimed to investigate which patient-reported activity is first associated with knee pain. We hypothesized that pain would occur first during activities requiring weight bearing and knee bending.

**Methods:**

Data were obtained from the Osteoarthritis Initiative (OAI), a multicenter, longitudinal prospective observational cohort of people who have or are at high risk of OA. Participants completed the Western Ontario and McMaster Universities Osteoarthritis Index (WOMAC; Likert scale) annually for up to 7 years. Rasch analysis was used to rank the WOMAC pain questions (activities) in order of affirmation as the pain score increased from 0. For each total WOMAC score category (0–20) we selected 25 individuals at random based on their maximum score across all visits. Fit to the Rasch model was assessed in this subset; stability of question ranking over successive visits was confirmed in the full OAI.

**Results:**

WOMAC data on 4,673 people were included, with 491 selected for subset analysis. The subset data showed good fit to the Rasch model (χ^2^ = 43.31, *P* = 0.332). In the full OAI, the “using stairs” question was the first to score points as the total pain score increased from 0 (baseline logit score ± 95% confidence interval −4.74 ± 0.07), then “walking” (−2.94 ± 0.07), “standing” (−2.65 ± 0.07), “lying/sitting” (−2.00 ± 0.08), and finally “in bed” (−1.32 ± 0.09). This ordering was consistent over successive visits.

**Conclusion:**

Knee pain is most likely to first appear during weight-bearing activities involving bending of the knee, such as using stairs. First appearance of this symptom may identify a group suitable for early intervention strategies.

## INTRODUCTION

The burden of osteoarthritis (OA) to the health sector in terms of cost (1), and the problems people have to endure with this condition are well known (2). Other than joint replacement, there is currently a lack of effective therapeutic interventions to structurally modify OA disease (3) and most treatment is mainly for symptomatic pain relief (4). Early detection of OA may enable effective interventions before major structural damage has occurred.

Pain is an important symptom associated with knee OA and this may be a good marker of population burden (5). Although structural biomarkers such as cartilage thickness may predict onset of pain (6), the effect of biomechanical loading on the joint may initiate the structural damage leading to OA (7). The role of biomechanical factors in the initiation of knee OA has been described (8). Although articular chondrocytes may adapt to changes in loading, prolonged mechanical stress leads to them failing (9). In people with unilateral hip OA it has been shown that there is a >2-fold increase in the likelihood of them developing OA in the contralateral knee (10). Asymmetrical loading of the joint has been observed in asymptomatic contralateral knees of those with hip OA, and this supports the notion that changes in dynamic load precede knee pain and knee OA (11). Apart from joint loading alone, which has been shown to be associated with changes in articular cartilage, repetition of loading may incite joint pain, and therefore measurement of both frequency and magnitude should be considered when assessing incidence of OA (12). Studies evaluating pain and occupation report that activities such as kneeling or squatting are linked to structural OA and the reporting of pain is greater in people whose occupation involves these activities (13).

In current clinical practice, conventional radiographs are known to be insensitive to OA structural changes, irrespective of symptoms (14). They are useful in measuring late-stage disease, but at this point surgery is often the only effective solution. Following knee pain onset, while radiographic measures have been shown to be useful in aiding subsequent OA diagnosis, no structural changes are seen early in most people (15). A long-term study of patients with chronic knee pain in the absence of radiographic features of OA found that the cumulative incidence of radiographic OA over a 12-year period was between 50% and 86%, depending on the cutoff used (Kellgren/Lawrence grade >1 or >0) (16).

Examining the onset of pain would contribute to what is known about early markers of OA. The Western Ontario McMaster Universities Osteoarthritis Index (WOMAC) is one of the most widely used measures to evaluate clinically important changes in people with OA and has been well validated (17). The WOMAC comprises 3 subscales measuring stiffness, pain, and function. The pain subscale questions relate pain to 5 different activities (walking, using stairs, lying in bed at night, sitting/lying down, and standing), each of which potentially inflicts a different mechanical joint loading. Identifying the activities during which symptoms first appear may therefore help indicate when structural damage first occurs, thereby permitting early diagnosis of OA to be made. This information would help target early intervention, perhaps increasing the efficacy of existing therapies and/or facilitating the development of novel treatments; it could also potentially aid in the screening of future participants for OA clinical trials.

The WOMAC has been shown to fit the Rasch model (18), which assesses the extent to which a scale displays a number of fundamental properties of true measurement. One of these is stochastic ordering of questions; different questions should represent different amounts of the latent quantity measured by the scale (different “item difficulties”) and some are, therefore, more likely to be affirmed before others as the level of the latent quantity increases. Rasch analysis therefore allows the examination of spacing of questions along a scale (19), providing a means of formally assessing which question, and hence which activity, is associated with the onset of pain. The objective of our study was to evaluate which of the activities captured by the WOMAC tended to become painful first as the pain score increased from 0, using Rasch analysis to compare the “locations” of the questions along the scale. We hypothesized that questions about activities that involve weight-bearing bending of the knee (using stairs, walking) would be the first to be affirmed.

**Significance & Innovations**

- Our findings indicate that the incidence of pain on stairs could be used to identify those who are first developing osteoarthritis knee pain, and this has implications for clinical practice.
- Our findings could help boost efficacy of interventions if symptomatic individuals were identified earlier.
- Our findings are of potential use in screening people for clinical trial eligibility.

## PATIENTS AND METHODS

### Data from the Osteoarthritis Initiative (OAI)

Data used in the preparation of this article were obtained from the OAI database, which is available for public access at http://www.oai.ucsf.edu/. Specific data sets used were AllClinical00 0.2.2; AllClinical01 1.2.1; AllClinical02 2.2.2; AllClinical03 3.2.1; AllClinical04 4.2.1; AllClinical05 5.2.1; AllClinical06 6.2.2; AllClinical07 7.2.1; AllClinical08 8.2.1; AllClinical09 9.2.1; and Enrollees 20.

### Participants

In total there were 4,796 individuals in the OAI (1,390 with confirmed radiographic OA, 3,284 deemed at high risk of developing OA, and 122 healthy controls). We excluded the controls from our analyses. Participants completed the WOMAC at baseline and then annually up to 72 months. A minority also completed the questionnaire at 18 and/or 30 months.

### WOMAC pain subscale

There are 5 questions in the WOMAC that ask the person to describe the pain they have experienced during certain activities: during walking, using stairs, in bed, sitting or lying, and standing. There are 5 possible responses: none, mild, moderate, severe, and extreme. These are scored 0–4.

### Data collation

All WOMAC pain subscale responses for both knees were compiled for all available time points. The total pain subscale score was calculated at each visit. For each individual, at each visit, the score from the knee with the highest total pain score was selected. Data collation was performed in SPSS (IBM), release 21.0.0.1.

### Descriptive analysis

We identified patients in the incidence cohort who scored 0 on the WOMAC at baseline and went on to score >0 at a later time point; the first knee to score >0 was selected. Proportions of patients affirming each item at the point of first scoring >0 were calculated. A Cochran's Q test for related samples was used to compare proportions affirming the different items because some patients affirmed more than 1 at that point. We then restricted the analysis to patients who had affirmed just 1 item; a 1-sample chi-square test was used to assess whether the 5 questions were equally likely to be affirmed first.

### Rasch analysis

#### Item and threshold locations

Rasch analysis relates the probability that a person will affirm an item (question) to the difference between the amount of the underlying trait they possess and the amount captured by the item. If a particular activity is only likely to be painful for those with moderate symptoms, those with mild symptoms should be unlikely to affirm that question, while those with severe symptoms should be almost certain to affirm it. The trait value captured by an item is expressed in logits.

Each item in the WOMAC has 5 response categories and therefore 4 associated thresholds. The thresholds are the points of transition on the logit scale immediately above or below which the degree of underlying pain will result in an individual giving a different response (the first threshold should correspond to the lowest degree of pain experienced, where the most likely response ceases to be “none” and shifts to “mild,” for example). The overall item location is determined by the mean of the individual threshold locations. We sought to identify the item that contained the threshold with the lowest logit score of all, representing the point of transition from a total pain score of 0 to a score of >0, i.e., the onset of knee pain.

#### Sample size

The fit statistics used in Rasch analysis assume an approximate chi-square distribution; errors in this approximation are trivial for most samples but can result in misfit in large samples (20). Poor targeting of the population of interest can also yield inaccurate estimates of item location (21). In order to obtain an accurate assessment of model fit, we selected a subset of participants, taking an equal number of individuals for each possible total pain subscale score category, therefore producing a sample where person ability and item difficulty could be estimated with an equal degree of precision across the scale. The highest score categories were used relatively rarely; therefore, the maximum total score recorded at any point during followup was calculated and used as a basis for selecting individuals for the assessment of fit to the Rasch model.

Rasch analysis requires a minimum sample size of 250 cases, or 20 times the number of items, whichever is greater, to produce sufficiently accurate estimates of item difficulty (99% + confidence) if the assessments will contribute to clinical diagnosis. Item difficulty estimates are likely to be free of substantive error for sample sizes approaching 500 (21). There are 21 possible WOMAC pain scores ranging from 0 to 20; therefore, we aimed to select 25 individuals for each, to give an intended sample size of 525. Frequencies of the maximum total pain scores were examined; if 25 or fewer participants had scores available for a given category, all of those individuals were selected. Where more than 25 participants had scores available, 25 were selected at random from the pool of individuals sharing that same total score.

#### Assessing fit to the Rasch model

Rasch analysis was conducted using RUMM2030. To assess fit to the Rasch model we checked for disordered item thresholds, item misfit (|fit residual|)>2.5, statistically significant chi-square and/or analysis of variance [ANOVA] tests of misfit), differential item functioning (DIF), local dependency, and multidimensionality. Finally we required a nonsignificant chi-square test of item–trait interaction to demonstrate good model fit. These tests have all been described in detail elsewhere (19). To assess the data for differential item functioning, the “person factors” of age (above/below median), sex, cohort (progression/incidence), and visit were included.

#### Assessing stability of item/threshold location

Once fit was confirmed in the subset, we identified the item with the smallest SE associated with the estimate of its location; the threshold locations estimated for this item in the subset analysis were used to anchor the data in the full sample. This ensured that the results at each visit in the full sample were calibrated to the same “ruler,” allowing item locations to be accurately compared, while allowing the locations of the other items to vary between visits.

## RESULTS

A description of the sample is shown in Table 1. The mean ± SD age was 61 ± 9.2 years (range 45–79 years). The majority were female (58%) and only 12% of the participants were reportedly seeing a health care professional for symptoms of arthritis.

**Table 1:** Characteristics of individuals in the incidence and progression cohorts of the OAI (n = 4,674)*

Characteristic	Result
Age, mean ± SD (range) years	61.3 ± 9.2 (45–79)
Age group, years	
45–54	1,346 (29)
55–64	1,525 (33)
65–74	1,370 (29)
≥75	433 (9)
Women	2,729 (58)
WOMAC pain (0–20), median (IQR)	
Baseline	2 (0–6)
Maximum[Table-fn tf1-1]	5 (3–9)
BMI, mean ± SD kg/m^2^	28.7 ± 4.8
BMI ≥30 kg/m^2^, no./total no. (%)	1,765/4,670 (38)
OAI cohort	
Incidence	3,284 (70)
Progression	1,390 (30)
Seeing HCP for arthritis	566 (12)

* Values are the number (percentage) unless indicated otherwise. OAI = Osteoarthritis Initiative; WOMAC = Western Ontario and McMaster Universities Osteoarthritis Index; IQR = interquartile range (first and third quartile); BMI = body mass index; HCP = health care professional.

† Recorded at any visit.

In the combined incidence and progression cohorts, 4,674 individuals completed the WOMAC at least once. Participants completed the questionnaire for each knee separately; in total there were 59,101 observations of WOMAC pain. When selecting a subset of individuals on the basis of the maximum score observed in either knee at any time point, for score categories 0–17 there were sufficient observations available to select 25 at random. For the higher scores, all available observations were selected (n = 14 for score category 18, n = 8 for score category 19, and n = 19 for score category 20), giving a subset sample size of 491.

### Descriptive analysis results

There were 550 patients in the incidence cohort with a complete set of WOMAC observations who scored 0 at baseline and went on to score >0. At the point of scoring >0, the proportions of patients affirming questions 1–5 were 40%, 81%, 19%, 21%, and 27%, respectively (Cochran's Q = 647.0, 4df, *P* < 0.001). Post hoc tests, adjusted for multiplicity, indicated that the proportion affirming question 2 was higher than for all other questions (*P* < 0.001 for all), and that the proportion affirming question 1 was higher than for questions 3–5 (*P* < 0.001 for all). Further restricting the analysis to the 280 patients who had affirmed just 1 question at this point, the proportions affirming questions 1–5 were 8%, 76%, 7%, 6%, and 4%, respectively (1-sample χ^2^ = 544.7, 4df, *P* < 0.001). Therefore, question 2 (using stairs) was apparently more likely to be the first affirmed. However, due to the impracticalities of examining further trends in the ordering of items and specific responses in the descriptive data, we proceeded with Rasch analysis to gain more insight.

### Fit to the Rasch model

The partial credit version of the Rasch model was chosen after a likelihood ratio test indicated that the assumption of the rating scale model (of equidistance of thresholds) was not supported (χ^2^ = 65.3, 11df, *P* < 0.001). All items exhibited ordered response thresholds, i.e., the thresholds between the score categories were found to be located in the expected order on the logit scale representing the underlying trait (pain in this case). Table 2 presents summary measures for model fit for the subset analysis. All absolute fit residuals were <2.5, and all chi-square and ANOVA tests of item misfit were nonsignificant following Bonferroni correction. There was no evidence of DIF by age, sex, cohort (progression, incidence), visit, or included knee (left or right).

**Table 2:** Summary measures of Rasch model fit in the subset of patients selected to ensure equal precision across the scale (n = 491)*

	Location	SE	Residuals	χ^2^	*P*	F†	*P*
Item							
1. Walking	−0.054	0.070	0.233	3.19	0.921	0.48	0.872
2. On stairs	−1.048	0.072	0.253	9.38	0.311	1.48	0.163
3. In bed	0.339	0.067	1.996	9.55	0.298	1.24	0.276
4. Lying/sitting	0.540	0.070	−0.534	12.83	0.118	2.03	0.041
5. Standing	0.223	0.070	−1.115	8.36	0.399	1.53	0.145
Mean ± SD	0.000 ± 0.624		0.167 ± 1.172				
Persons							
Mean ± SD	−0.270 ± 2.451		−0.445 ± 1.160				
Model fit							
Item–trait interaction PSI = 0.90/0.89‡				43.31	0.332		

* PSI = person separation index.

† For analysis of variance F statistic.

‡ Excluding individuals with “extreme” scores (at the very top or bottom of the range).

There was no substantive local dependency among the 5 items. However, they showed evidence of slight multidimensionality; the principal component analysis revealed 3 negatively loading items (1, 2, and 5, i.e., walking, stairs, and standing, respectively) and 2 positively loading items (3 and 4, i.e., in bed and while lying or sitting, respectively). The lower 95% confidence limit for the number of significant *t*-tests comparing person scores using the negatively or positively loading questions was 6.3%; values above 5% indicate multidimensionality. However, it is recommended that item subsets selected for the *t*-tests provide at least 12 thresholds in each group. This is not possible for the WOMAC pain subscale, as there are only 20 thresholds available (4 for each of 5 items). Subtesting (combining) items 1, 2, and 5 together and items 3 and 4 together to produce 2 “testlets” or superitems, then reevaluating the results indicated that the 2 putative dimensions in fact shared 92% of the nonerror variance, supporting a strong unidimensional construct. The internal consistency and reliability of the subset analysis were very good (person separation index = 0.89). Overall there was good fit to the Rasch model (χ^2^ = 43.31, *P* = 0.332).

### Targeting of the scale

**Figure 1:**
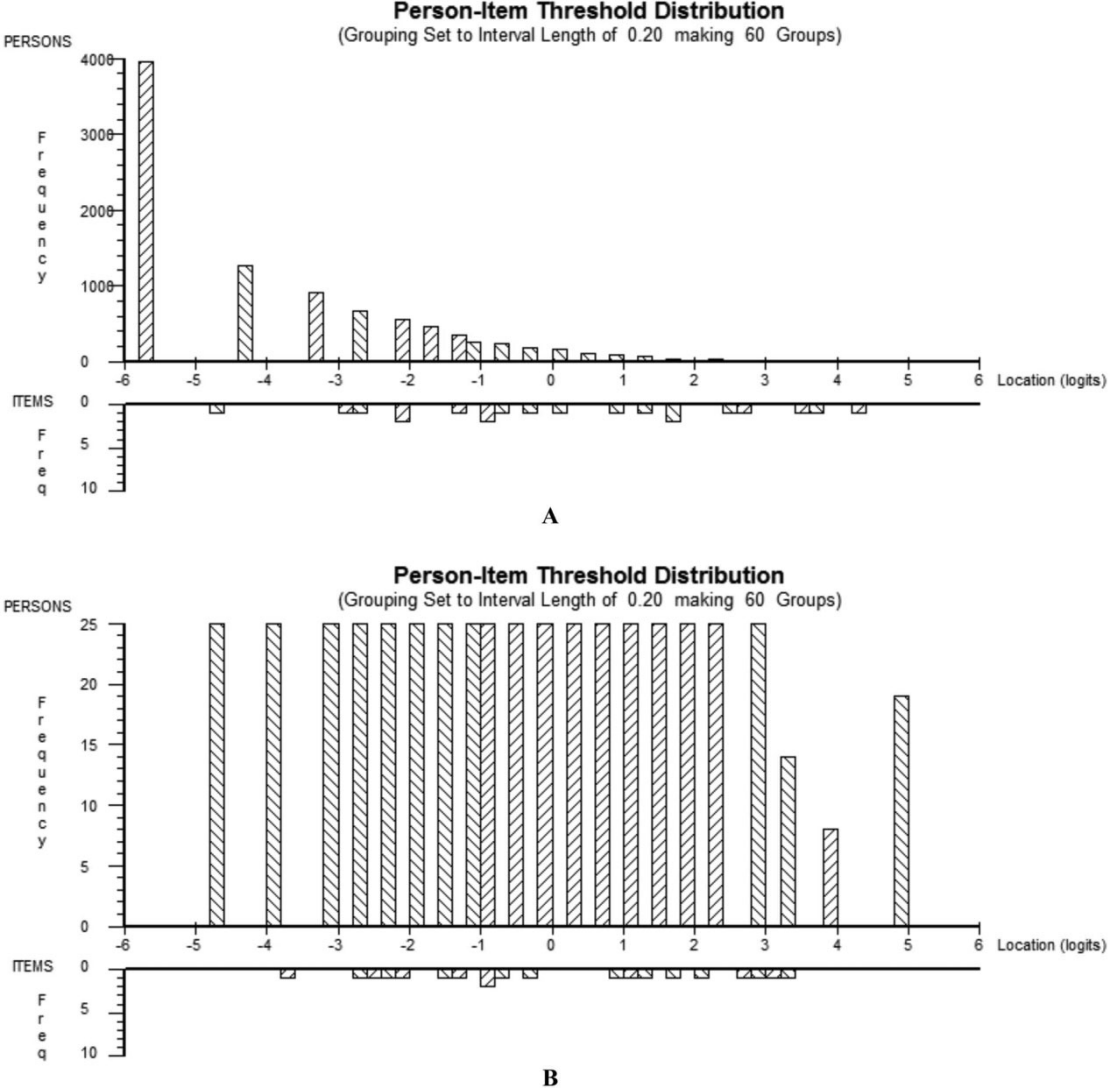
The person-item threshold distribution in the total sample (A; n = 9,348 persons [knees], baseline score in both knees included) and in the subset of patients selected to ensure equal precision across the scale (B; n = 491 persons, single knee with maximum score observed at any time included).

The distribution of item thresholds and person scores in the combined incidence and progression cohorts at baseline and in the subset (for whom the maximum score observed at any time point was selected) are shown in Figure 1. It is clear that in the total sample at baseline the “persons” were clustered toward the bottom end of the scale (the majority of pain scores were low), so they were not well targeted by the item thresholds. In the subset there was complete overlap between the person abilities and the threshold locations, as expected given the selection process.

### Item and threshold locations

In the subset analysis, the overall item location (the mean of the threshold locations) for the question “pain on going up or down stairs” was considerably lower than for the other items (−1.048 versus −0.054 to 0.540), indicating that on average this item tended to be affirmed before the others. However, only inspection of the individual threshold locations could confirm which item (and specifically which transition threshold across categories) captured the transition from “no pain” to “pain.”

**Figure 2:**
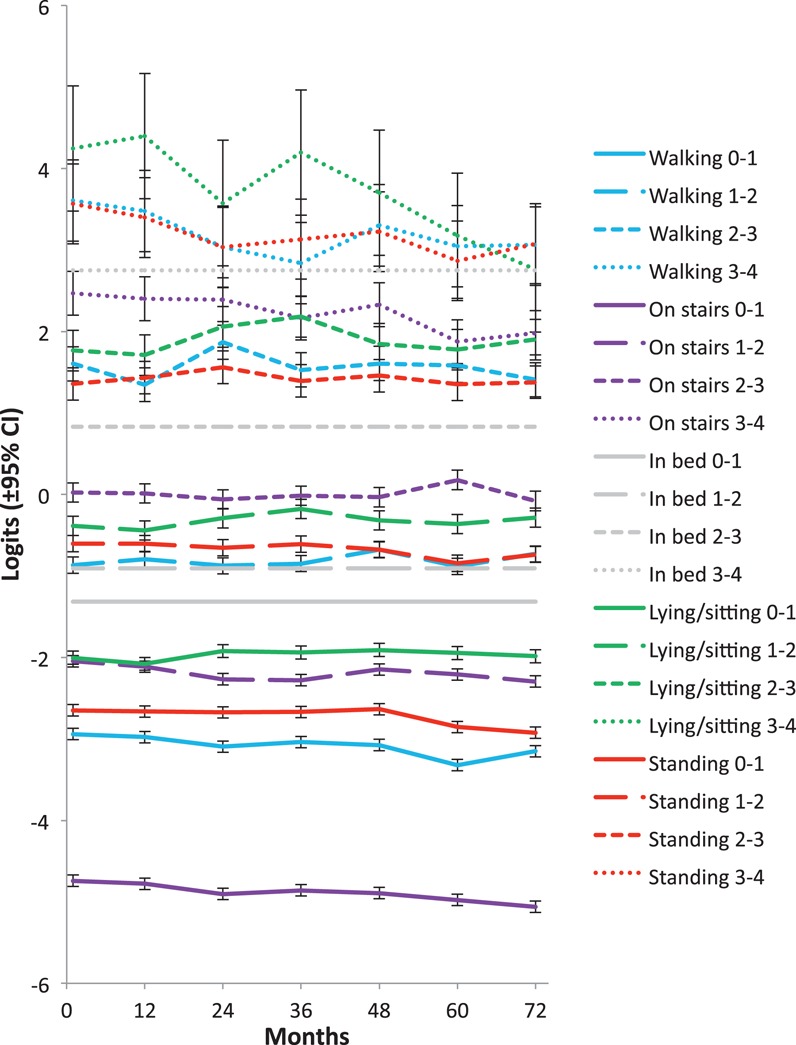
The distribution of the threshold locations (logit ± 95% confidence interval [95% CI]) from the 5 Western Ontario and McMaster Universities Osteoarthritis Index items in the total sample (n = 4,674), anchored to the threshold locations estimated for “pain when lying in bed,” in the subset of patients selected to ensure equal precision across the scale, at successive annual visits up to 72 months. 0–1 = threshold between none and mild; 1–2 = threshold between mild and moderate; 2–3 = threshold between moderate and severe; 3–4 = threshold between severe and extreme.

Individual threshold locations for all items in the full sample, at each visit, are presented in Figure 2. For the highest thresholds (the transition points between the responses “severe” and “extreme” as indicated by the smallest dotted lines), the confidence intervals were wide and the location estimates varied considerably from visit to visit, reflecting the fact that relatively few participants gave these responses at each visit. By comparison, because the majority of individuals were clustered toward the lower scores, and therefore the lower thresholds, the estimates of the lower threshold locations were very accurate and much more consistent over time. At baseline the first threshold for the stairs question was at −4.7 logits (±0.07); the next lowest threshold for the first for the walking item, which at −2.9 logits (±0.07) was almost 2 logits higher on the scale. Therefore, the results clearly showed that the first threshold for the stairs item had the lowest logit score of all, by a considerable margin, and this finding was consistent across all visits in the full sample. The order in which the items started to score points as total pain score increased from 0 was “pain using stairs” > “pain during walking” > “pain while standing” > “pain while sitting or lying” > “pain in bed.” This supports the hypothesis that pain first appears during activities that involve weight-bearing bending of the knee.

## DISCUSSION

In a large observational cohort of people with confirmed radiographic knee OA, or who were considered at high risk of developing knee OA, Rasch analysis showed that, of 5 activities that may result in different mechanical loadings on the knee joint, using stairs was most likely to be the first to cause pain. This is consistent with the hypothesis that activities involving weight-bearing bending of the knee are the first to become painful as OA develops, and suggests that, in order to identify those at the earliest stages of OA who may benefit from early intervention, individuals in high risk groups should be monitored for the development of pain during such activities.

This is not the first time Rasch analysis has been used to assess the WOMAC, although to our knowledge it is the first study to use the results to help determine which activities might be associated with the onset of knee pain. Previous work has focused on the validity of the scale itself in different settings; however, the results were consistent with our findings. Wolfe and Kong (18) administered the visual analog scale version of the WOMAC to patients with OA, rheumatoid arthritis, or fibromyalgia; in each patient group the stairs item was associated with the lowest logit score. Davis et al (22) also found that the stairs item was consistently associated with the lowest logit score, whether in a community sample reporting hip or knee complaints or in a surgical sample of patients awaiting total knee or hip replacement, both pre- and postoperatively. Ryser et al (23) administered the 0–10 numeric rating scale version of the WOMAC to OA patients and performed an analysis that included all 24 WOMAC items; the stairs item was not only the pain item with the lowest logit score, it had the lowest logit score of any item in the full WOMAC. Therefore, pain on stairs may be the first symptom to be experienced (of those captured by the WOMAC). However, because the item difficulty is just the average of thresholds, the item with the lowest logit score is not guaranteed to contain the threshold with the lowest logit score; none of these studies reported individual threshold locations.

Our results show that the order in which other activities captured by the WOMAC became painful was consistent with the hypothesis that weight bearing, particularly bending of the knee, is associated with pain onset; the next question to score points after “using stairs” was “walking” (involving both weight bearing and bending), then “standing” (weight bearing, but no bending) followed by “lying/sitting” (no weight bearing, some bending involved) and lastly “in bed” (neither weight bearing nor bending). Pain experienced while lying or sitting, or while in bed, is perhaps less likely to have a mechanical origin. It is tempting to speculate as to whether pain during these activities might be the result of underlying structural damage within the bone itself.

There are limitations to this study. Despite good overall fit to the Rasch model, there was some evidence of slight multidimensionality. However, there was limited ability to accurately assess dimensionality because, due to the nature of the scale, we did not have the recommended sample size of 12 thresholds for the comparison between positively and negatively loading items. Subtest analysis suggested that there was little “unique” information coming from each of the 2 putative dimensions, as adding them together accounted for 92% of the nonerror variance on the underlying construct. It is perhaps itself interesting that the negatively loading items (walking, stairs, and standing) all involved weight bearing, while the positively loading items (lying/sitting, in bed) did not. We feel it unlikely that this slight multidimensionality could have greatly affected our conclusion that pain first appears during weight-bearing activities such as using stairs.

Another potential limitation is the use of the WOMAC itself, which captures self-reported pain during activities rather than obtaining symptom measures related to observed performance-based testing. Subjective interpretation of the questions could lead to variable results; however, pain will always be subjective irrespective of the nature of the associated activity. For early intervention programs, including people with easily recognizable activity-related symptoms, rather than performance-related measures, will be feasible. The extent of subjective differences in interpretation was limited in our sample, as the Rasch model tests for this by examining fit of persons to the model expectations, and only 5 individuals out of 491 (1%) had very poor fit, indicating response patterns significantly different to the sample as a whole.

We included individuals from both the incidence and progression cohorts in our Rasch analyses; this meant that some did not have radiographically confirmed OA. However, we found no evidence of DIF by cohort, indicating that the same pattern of responses was seen in each, and therefore that the stairs item was consistently the first to be affirmed, irrespective of whether or not patients had radiographic OA.

This study focused on patient-reported, activity-related pain. It is possible that other types of pain may precede the development of activity-related pain. However, 2 of the questions in the WOMAC capture pain while inactive (pain while lying/sitting and pain in bed), and these were the last to be affirmed, so giving empirical support to activity-related pain appearing first.

The descriptive longitudinal data from the incidence cohort supported the findings of the Rasch analysis; however, existing frequent knee symptoms were major risk factors determining eligibility for the study, hence patients selected for analysis on the basis that they scored 0 on the WOMAC at baseline might have scored >0 at some point in the preceding year. Our Rasch analysis helped us to formally identify the transition point between absence and presence of pain, which on a conceptual basis we have taken to be the point of pain onset. However, gathering empirical evidence that pain would first develop when using stairs would involve a long-term, stand-alone prospective study of people with no symptoms at baseline.

In conclusion, this study shows that knee pain is most likely to first appear during weight-bearing activities involving bending of the knee, such as using stairs. Prospective trials will help to determine whether people who develop OA can be identified sooner if pain during such activities is used during screening, perhaps facilitating effective intervention to prevent further progression of the disease.

## AUTHOR CONTRIBUTIONS

All authors were involved in drafting the article or revising it critically for important intellectual content, and all authors approved the final version to be submitted for publication. Dr. Hensor had full access to all of the data in the study and takes responsibility for the integrity of the data and the accuracy of the data analysis.

**Study conception and design.** Hensor, Dube, Kingsbury, Conaghan.

**Analysis and interpretation of data.** Hensor, Dube, Kingsbury, Tennant, Conaghan.

## ROLE OF THE STUDY SPONSOR

The Osteoarthritis Initiative private funding partners (Merck Research Laboratories, Novartis Pharmaceuticals Corporation, GlaxoSmithKline, and Pfizer) had no role in the study design, data collection, data analysis, or writing of this manuscript. Publication of this article was not contingent on the approval of these sponsors.
